# Impact of *Leishmania donovani* infection on the HLA I self peptide repertoire of human macrophages

**DOI:** 10.1371/journal.pone.0200297

**Published:** 2018-07-12

**Authors:** Lydon Wainaina Nyambura, Saulius Jarmalavicius, Peter Walden

**Affiliations:** 1 Charité –Universitätsmedizin Berlin, corporate member of Freie Universität Berlin, Humboldt-Universität zu Berlin, and Berlin Institute of Health, Department of Dermatology, Venerology and Allergology, Clinical Research Group 'Tumor Immunology', Berlin, Germany; 2 Humboldt Universität zu Berlin, Lebenswissenschaftliche Fakultät, Institut für Biologie, Berlin, Germany; Hospital Israelita Albert Einstein, BRAZIL

## Abstract

Macrophages are specialized antigen-presenting cells that process and present self-antigens for induction of tolerance, and foreign antigens to initiate T cell-mediated immunity. Despite this, *Leishmania donovani* (LD) are able to parasitize the macrophages and persist. The impact of this parasitizing and persistence on antigen processing and presentation by macrophages remains poorly defined. To gain insight into this, we analyzed by liquid chromatography tandem mass spectrometry (LC-MS/MS) and compared the HLA-I self-peptidomes, proteasome compositions, HLA expression and activation states of non-infected and LD-infected THP1-derived macrophages. We found that, though both HLA-I peptidomes were dominated by nonapeptides, they were heterogeneous and individualized, with differences in HLA binding affinities and anchor residues. Non-infected and LD-infected THP1-derived macrophages were able to sample peptides from source proteins of almost all subcellular locations and involved in various cellular functions, but in different proportions. In the infected macrophages, there was increased sampling of plasma membrane and extracellular proteins, and those involved in immune responses, cell communication/signal transduction and metabolism/energy pathways, and decreased sampling of nuclear and cytoplasmic proteins and those involved in protein metabolism, RNA binding and cell growth and/or maintenance. Though the activation state of infected macrophages was unchanged, their proteasome composition was altered.

## Introduction

Visceral leishmaniasis (VL) is a vector-borne neglected tropical and subtropical disease caused by *Leishmania donovani* (LD) and other *Leishmania* species that are transmitted by infected female phlebotomine sandflies and obligate intracellular protozoan parasites in vertebrates [1]. It is the second largest parasitic killer disease after malaria, with 200 000 to 400 000 estimated new cases each year. The infections result in high fatality [1–3] that is prone to worsen with HIV/VL and other co-infection [4–7].

When promastigote forms of LD are taken up by the macrophages they are internalized into phagolysosomes where they transform into non-motile amastigotes. The amastigotes survive the harsh milieu in the phagolysosome, multiply, and eventually rupture the macrophages and infect the new macrophages [8]. Survival and replication in the macrophage host is through subversion of the host immune system and promotion of pro-parasitic host factors [9]. This is achieved, among other mechanisms, by inhibiting the formation of nitric oxide that plays a major role in killing intracellular parasites, suppressing apoptosis of the host cell, and inhibiting production of cytokines thus interfering with cytokine-inducible macrophage functions such as oxidative bursts [10–14].

Macrophages, as professional antigen-presenting cells, generate MHC class I and MHC class II (HLA class I and II in humans) peptide complexes through various intracellular pathways and mechanisms. For MHC I peptide complexes, endogenous or cross-presented proteins are processed via constitutive or immunoproteosomes. The resulting peptides are transported into the endoplasmic reticulum by the transporter associated with antigen processing (TAP), loaded onto MHC class I molecules (HLA I), and transported via the Golgi apparatus and exocytic vesicles to the cell surface for presentation to CD8^+^ T cells [15]. The collection of peptides presented by MHC molecules at the cell surface are termed HLA peptidomes or ligandomes. Though LD is able to parasitize the host macrophages and persist, its impact on the host HLA-I peptidome remains undefined.

The human monocytic leukemia cell line THP1-derived macrophages (THP1MФ) are similar to native monocyte-derived macrophages [16–19], and have been used extensively as model for studying human macrophage immune functions and responses towards intracellular pathogens [19, 20]. The THP1MФ could therefore serve to study and compare the self-peptidomes of human macrophages infected and non-infected with LD, which would allow to gain insights into how LD influences antigen processing and presentation.

## Material and methods

### Parasites

The wild type LD MHOM/IN/02/BHU5 (BHU5) [21] promastigotes were established from splenic aspirates of an Indian patient and cultured in M199 culture medium (Gibco Invitrogen) supplemented with 20% heat-inactivated fetal bovine serum (Biochrom, Germany) at 25°C. The yellow fluorescent protein (YFP) transfected LD (YFP-BHU5) promastigotes were cultured in the dark in the same medium with 50μg/ml hygromycin.

### Cell lines, differentiation and infection

The HeLa cell line clone 33/2 (HeLa A2+/IP) that stably expresses the three inducible subunits LMP2, MECL1 and LMP7 [22] was kindly provided by Prof. P. M. Kloetzel (Charité - Universtitätsmedizin Berlin, Germany). It was cultured in Iscove’s Basal medium (Biochrom Germany) supplemented with 10% heat-inactivated fetal calf serum (Biochrom, Germany), 2 μg/ml puromycin (Roche, Mannheim, Germany) and 300 μg/ml hygromycin (Roche, Mannheim, Germany) at 37°C in a humidified atmosphere with 8% CO_2_. The acute monocytic leukemia-derived human cell line THP1 (ATCC TIB-202) was cultured in RPMI 1640 medium (Invitrogen, Karlsruhe, Germany) supplemented with 10% heat-inactivated fetal calf serum (Biochrom, Germany) and 1% streptomycin-penicillin (Gibco Invitrogen, Germany) at the same conditions. THP1 is homozygous for HLA-A*02:01, HLA-B*15:11 and HLA-C*03:03 [23]. To generate the THP1 macrophage phenotype (THP1MФ), THP1 cells were differentiated with 50 ng/ml phorbol-12-myristate-13-acetate (Sigma, Steinheim, Germany) for 48h. To infect THP1MФ with the BHU5 or YFP-BHU5, parasites were harvested by centrifugation at 500 x g for 8 minutes, washed, re-suspended in RPMI 1640 medium (Invitrogen, Karlsruhe, Germany) and added to the THP1MФ cultures at the ratio parasites: THP1MФ 10:1, and cultured for 48 h at 37°C under 8% CO_2_. Subsequently, THP1MФ and YFP-BHU5 infected THP1MФ (THP1MΦiy) were used immediately to access the uptake of YFP-BHU5 by THP1MФ and its effects on HLA-ABC and HLA-DR expression. THP1MФ and BHU5 infected THP1MФ (THP1MΦi) were used to access viability, HLA-ABC, HLA-A*02:01 and CD83 expression, and were harvested by 10 min centrifugation at 800 x g, shock-frozen in liquid nitrogen and stored as pellets at -80°C for isolation of HLA.

### Parasite uptake, viability, activation and HLA expression

To determine the uptake of LD by THP1MΦ and effects on hosts viability, CD83, HLA-ABC, HLA-A*02:01 and HLA-DR expression flow cytometry was used. THP1MФ and THP1MΦiy or THP1MΦi were stained with fluorochrome-labeled monoclonal antibodies against CD11b, CD83, HLA-ABC (BD Bioscience, Heidelberg, Germany), HLA-A*02:01 and HLA-DR (BioLegend, Eching, Germany), Calcein-AM (Invitrogen, Carlsbad, CA, USA) and Propidium Iodide (PI) (Sigma-Aldrich, Germany). The expression of these markers on the cell surface and of calcein and PI fluorescence was determined with a FACSCalibur flow cytometer (Becton Dickinson, Heidelberg, Germany) on 20,000 for forward versus sideward scatter-gated events. CellQuest (Becton Dickinson, Heidelberg, Germany) and WinMDi 2.9 (Purdue University, USA) software were used to process and analyze the data, respectively. The uptake was assessed by CD11b expression against YFP fluorescence, and cell viability assessed using Calcein-AM (Invitrogen, Carlsbad, CA, USA) and propidium iodide (PI) (Sigma-Aldrich, Germany). CD83 was used as a marker of macrophage activation.

### Constitutive and immunoproteasome expression in THP1MФ and THP1MФi

The impact of the infection by LD on the constitutive and immunoproteasome expression of THP1MΦ was determined by semi-quantitative RT-PCR. Total RNA was extracted from THP1MФ, THP1MФi, HeLa cell line, and HeLa clone 33/2 (A2+/IP) using Nucleospin RNA II Purification Kit (Macherey-Nagel, Duren, Germany). cDNAs were prepared from 500ng of DNase-treated RNA using superscript III reverse transcriptase (Invitrogen, CA, USA). RT-PCR was carried out with 500ng of cDNA using the following constitutive (β1, β2 and β5) and immunoproteasome (β1i, β2i and β5i) subunit-, and GADPH- specific forward and reverse primers [24]. β1: GACTCCAGAACAACCACTG, CTTGGTCATGCCTTCCCG (399bp; BC000835.2, NM_057099.2); β2: CTGAAGGGATGGTTGTTGC, CTTTCTCACACCTGTACCG (558bp; D38048.1, NM_053532.1); β5: CCAAACTGCTTGCCAACATG, GAGTAGGCATCTCTGTAGG (275 bp; D29011.1, XM_341314.3); Hsβ1i: CTACTGTGCACTCTCTGG, GCCTGGCTTATATGCTGC (313 bp; U01025); Hsβ2i: GAAGATCCACTTCATCGC, CTCCAGGGTTAGTGGCTTC (571 bp; Y13640); Hsβ5i: GGAGAAAGGAACGTTCAG, TTGATTGGCTTCCCGGTAC (648 bp; U17496); GAPDH: CCTTCATTGACCTCAACTAC, CACCACCCTGTTGCTGTAG (869 bp; NM_002046.2, NM_017008.2). PTC-200 Peltier Thermal Cycler (BIO-RAD, München, Germany) was used. Thermocycling conditions were denaturation at 96°C for 2 min, 30 cycles of denaturation at 95°C for 40 sec, primer annealing at 55°C to 68°C for 1 min, primer extension at 72°C for 40 sec and a final cycle of extension at 72°C for 10 min. The amplified DNA fragments were analyzed by electrophoresis using 1.2% agarose gels. HeLa cell line, and HeLa clone 33/2 (A2^+^/IP) were used as positive controls for constitutive and immunoproteasome subunits, respectively. GelAnayzer2010 (http://www.gelanalyzer.com/download.html) was use to semi-quantitatively analyze the subunit band intensities. For each subunit, the band intensity was divided by the value for the GAPDH amplified in the same reaction tube.

### Isolation, purification and LC-MS/MS analysis of HLA I-presented peptides

Isolation of MHC I molecules was carried out as previously described [25, 26]. In brief, 2.3 x 10^9^ shock frozen cells were lyzed in 0.3% CHAPS, 0.2% NP-40, 145 mM NaCl, 1 mM EDTA, 1mM Pefabloc, 20 mM Tris-HCl buffer at pH 7.4 and ultracentrifuged for 1h at 100,000 x g. HLA I molecules were purified from the supernatants using monoclonal antibody of irrelevant specificity for preclearing and HLA class I-specific mAb W6/32, respectively, coupled to CNBr-activated sepharose (Amersham Biosciences, Uppsala, Sweden). The anti-human HLA-I column with HLA-peptide complexes was washed successively with 20mM Tris, 145 mM NaCl pH 7.4 (TBS), 0.3% CHAPS in TBS, TBS, 0.3% ß-octylglycoside in TBS, TBS and finally ultrapure water. HLA-peptide complexes were eluted using 0.7% TFA in ultrapure water. Peptides were isolated from high molecular weight components by ultrafiltration using centrifugal filters with a 3-kDa molecular weight cut-off (Centricon, Millipore, Schwalbach, Germany). Filtrate fractionates were obtained using an acetonitrile gradient of 5–90% of solvent B (90% acetonitrile, 0.1% TFA in ultrapure water) in solvent A (0.1% TFA in ultrapure water) with a reverse phase column μRPC C2/C18, SC2.1/10 on a Smart HPLC system (Amersham Biosciences, Freiburg, Germany). The peptide fractionates were analyzed by reverse phase liquid chromatography (3000 nano-HPLC system; Dionex, Darmstadt, Germany) coupled on-line with MicroTOF-Q mass spectrometer (Bruker Daltonics, Bremen, Germany). Peptide fractionates were injected onto a C18 precolumn at 20 μL/min (2% acetonitrile, 0.05% TFA) for 5 min. Subsequently, peptides were separated at a flow rate of 220nl/min onto a 75-μm × 15 cm PepMap nano-HPLC column with a gradient of 5–60% acetonitrile over 60 min, then 60–90% acetonitrile over 5 min and finally 90% acetonitrile for 5 min, all with 0.1% formic acid in ultrapure water. Eluted peptides were nanospray-ionized and fragmented based on the five most intense precursor ions signals, with a 1 min dynamic exclusion time to avoid repeated fragmentation.

### Processing and analysis of data

MS and MS/MS spectra were processed using Data Analysis 3.4 and Biotools 3.1 (Bruker Daltonics). Peptides were identified against Swissprot human protein sequence database version 56.3 (20,408 reviewed non-redundant protein sequences) integrated in a local MASCOT server (version 2.2). Precursor and fragment mass tolerances of 100 ppm and 0.5 Da were used respectively, oxidation of methionine was allowed as a possible modification. Peptide-spectrum matches were validated using a statistical evaluation -10logP, where logP is the logarithm to the base 10 of P (P<0.05). *De novo* sequencing using Sequit software [27] and manual inspection were used to further validate the identified peptides. Peptides source proteins were annotated using Uniprot [28] and classified according to subcellular locations and biological functions using human protein reference database [29]. Peptides were assigned to their respective HLA using netMHCpan in the Immune Epitope Database IEDB [30, 31] and SYFPEITHI [32], and their predicted HLA binding affinities were determined using netMHCpan in IEDB with a binding affinity IC_50_ threshold of 500nM. Binding motifs for the nonapeptides were visualized using sequence logos [33, 34].

### Statistical analyses

Macrophage activation, HLA expression and proteasome subunit expression between THP1MФ and THP1MФi/or THP1MФiy were compared by paired 1-tailed Student’s t-test and differences indicated as significant when *p < 0.05. Data are presented as the mean ± standard deviation from three independent experiments.

## Results

### Parasite uptake and effects on viability, activation and HLA expression

**Fig 1:**
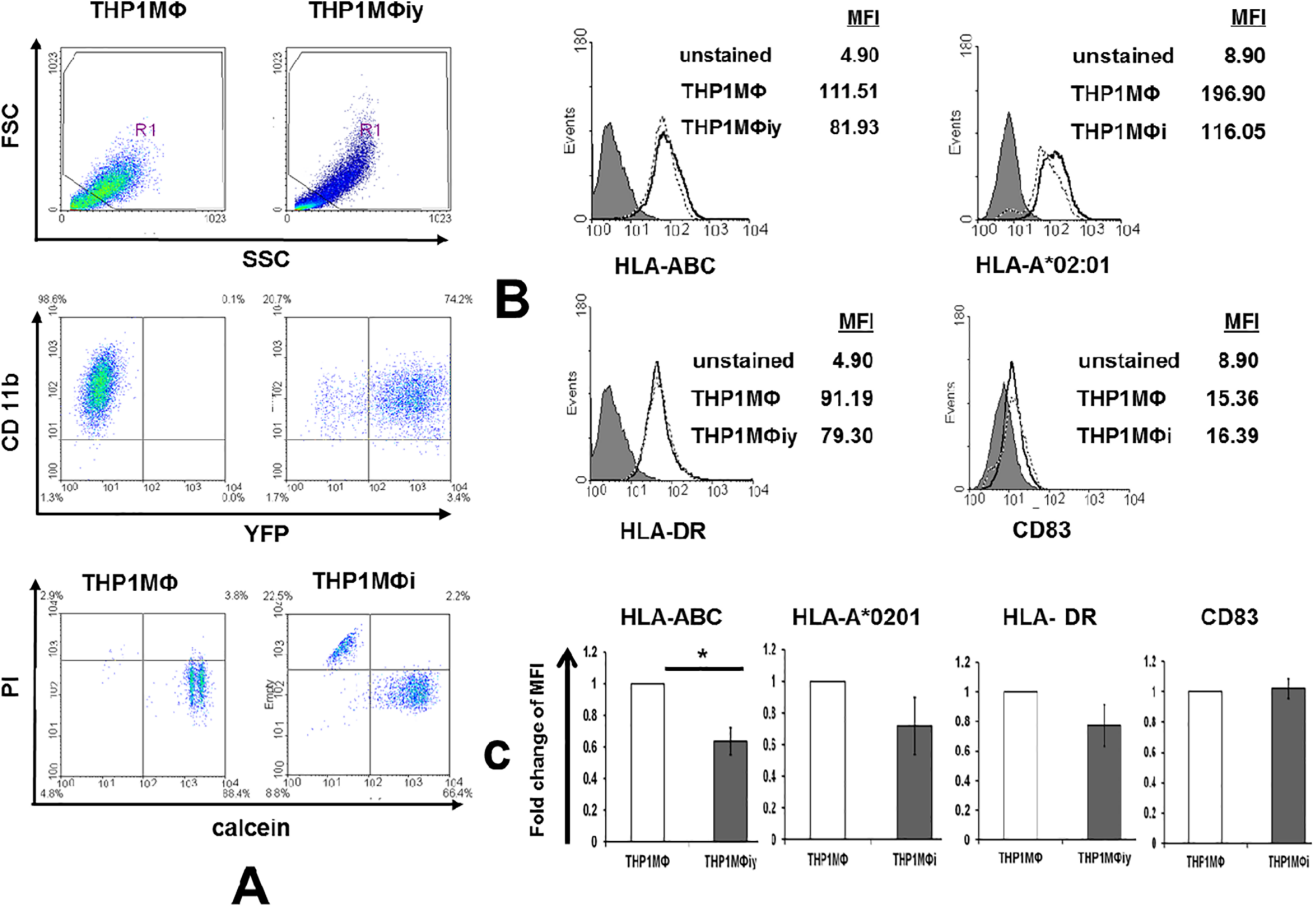
Uptake of *Leishmania donovani* by THP1MФ: effects on viability, and HLA-ABC, HLA-A*02:01, HLA-DR and CD83 expression. A) Flow cytometric analysis of the uptake of YFP-BHU5 by THP1MΦ assessed by the YFP fluorescence in CD11b expressing THP1MΦi compared to THP1MΦ, and viability of THP1MФ and THP1MФi analyzed by calcein and PI staining. B) Expression levels of HLA-ABC, HLA-A*02:01, HLA-DR and CD83 on THP1MΦ and THP1MФi or THP1MФiy. C) Representation of the expression levels of HLA-ABC, HLA-A*02:01, HLA-DR and CD83 as fold change of MFI in THP1Mɸi or THP1Mɸiy below/above that of THP1Mɸ. Error bars represent ±SD of the mean of three independent experiments; *p < 0.05 comparing THP1MΦ vs THP1MФi or THP1MФiy. Fig 1A and 1B are representatives of three independent experiments.

To assess the uptake of LD by THP1MΦ and to determine its effects on viability and expression of HLA-ABC, HLA-A*02:01, HLA-DR and CD83 by the host, flow cytometry was used as detailed in Materials and Methods. The uptake of YFP-BHU5 by THP1MФ based on CD11b expression versus the YFP fluorescence was 70.25±5.59%. The viability of THP1MФi was slightly lower compared to THP1MФ based on calcein and PI fluorescence **(Fig 1A)**. The levels of HLA-ABC (*p<0.05), HLA-DR and HLA-A*02:01 were lower on the infected compared to the non-infected cells, while those of the macrophage activation marker CD83 were unchanged **(Fig 1B and 1C)**. The decrease in MHC class I and II expression on infected cells has also been observed previously, albeit in murine studies [14].

### Impact of parasite uptake on constitutive and immunoproteasome mRNA expression

**Fig 2:**
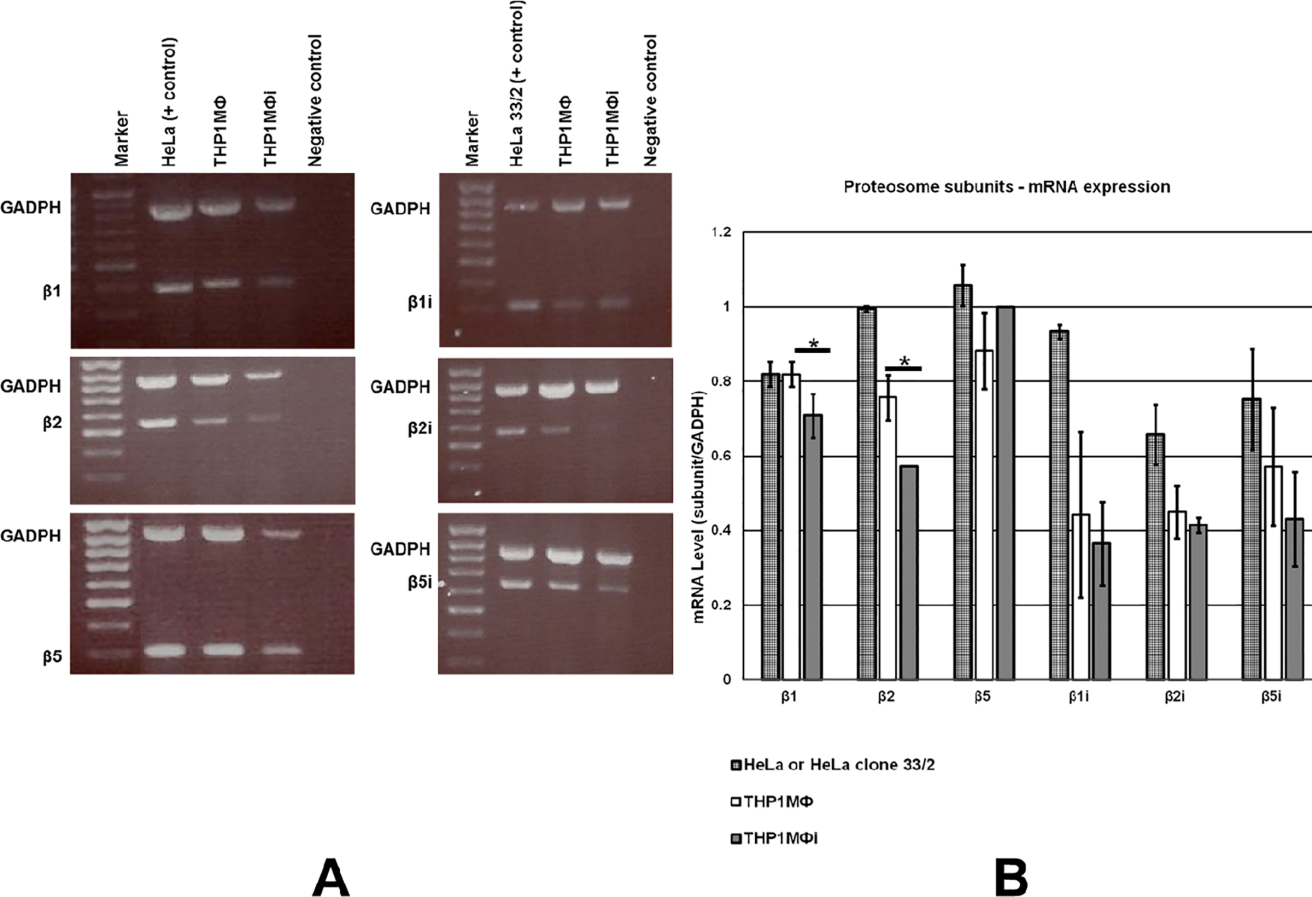
mRNA expression levels of constitutive proteasome subunits β1, β2 and β5 and the immunoproteasome subunits βi1, βi2 and βi5 in THP1MФ and THP1MФi. A) Agarose gel electrophoresis of RT-PCR of the proteasome subunits β1 (399bp), β2 (558bp) and β5 (275bp) and the immunoproteasome subunits βi1 (313bp), βi2 (571bp) and βi5 (648bp) in THP1MФ and THP1MФi, HeLa (positive control for constitutive proteasome subunits) and HeLa clone 33/2 (positive control for immunoproteasome subunits), and GADPH as internal control. B) Shows mean ± SD of three independent experiments of semi-quantified mRNA expression of proteasome subunits normalized to GADPH mRNA expression in the same reaction using GelAnalyser 2010. *p < 0.05 comparing THP1MΦ vs THP1MФi.

To determine the impact of LD on the hosts constitutive and immunoproteasome subunit expression by THP1MΦ, semi-quantitative RT-PCR was carried out as detailed in Materials and Methods. THP1MФ and THP1MФi expressed both the constitutive proteasome subunits (β1, β2 and β5) and the immunoproteasome subunits (βi1, βi2 and βi5) **(Fig 2A)**. Semi-quantitative analysis of the RT-PCR bands showed a reduction of the mRNA (subunit/GADPH) of β1 (*p<0.05), β2 (*p<0.05), and no significant change for the β2i (p = 0.28) and β5i (p = 0.15) subunits in THP1MФi compared to THP1MФ with 14%, 25%, 9% and 25%, respectively **(Fig 2B)**.

### Self-ligands presented by HLA I of THP1MФ and THP1MΦi

2.3 x 10^9^ THP1MΦi cells were lysed, and affinity chromatography and LC-MS/MS was used to isolate the HLA class I molecules and analyze the HLA-bound peptides. A total of 86 non-redundant HLA class I self-ligands were identified from 82 source proteins of THP1MФi **(S1 Table)** compared to 347 non-redundant HLA-I self-ligands derived from 282 source proteins identified for 2.8 x 10^9^ THP1MΦ cells at the same time and reported earlier [26]. Only 17 HLA-I self-peptide sequences and 18 source proteins were found to be shared between THP1MФ and THP1MФi.

### HLA I-bound peptide lengths

**Fig 3:**
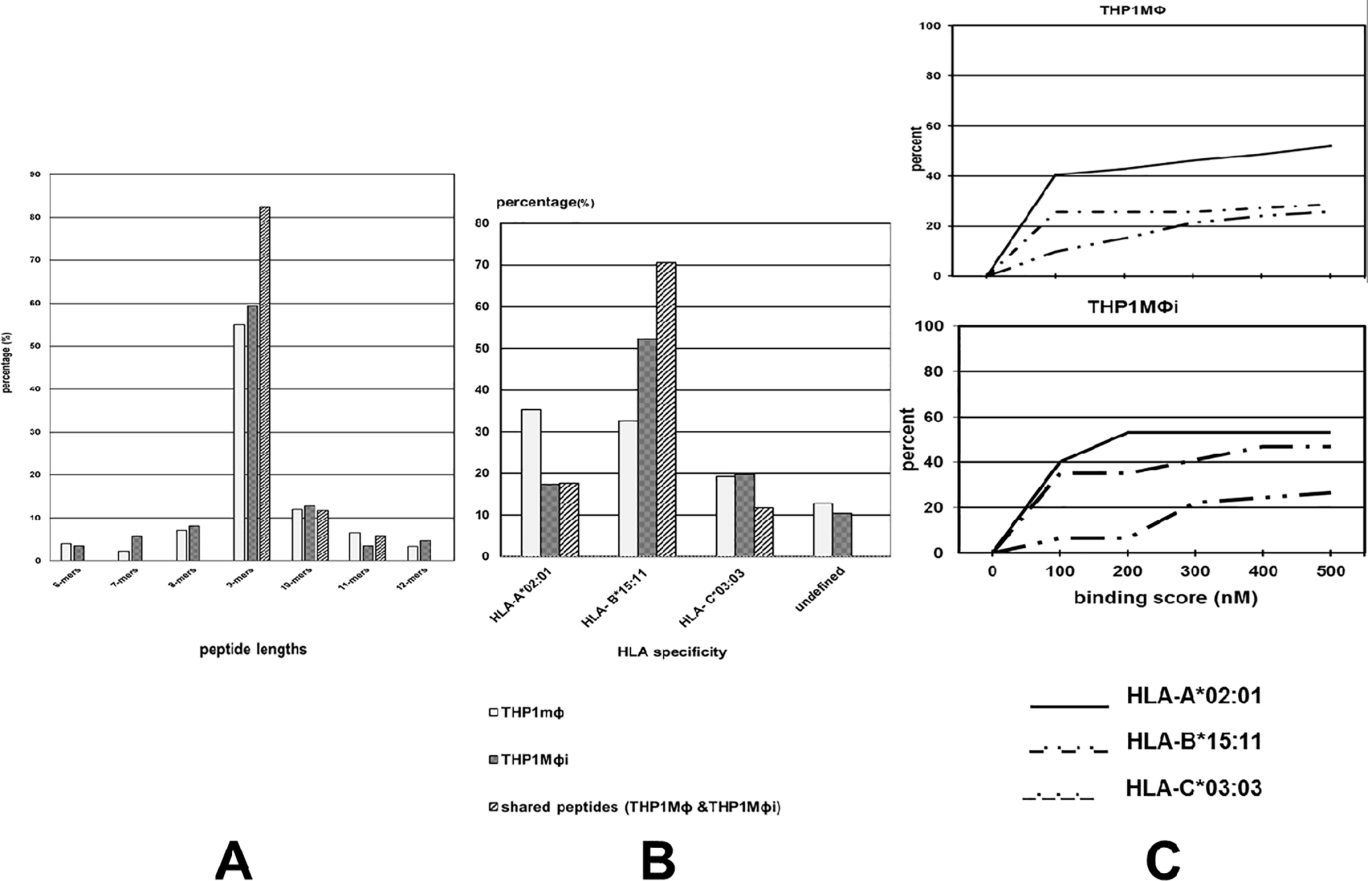
HLA class I self-peptide lengths, HLA restriction and binding affinities. A) HLA Class I peptide lengths in THP1MΦ and THP1MΦi. B) MHC restriction of HLA I-bound self-peptides from THP1MΦ and THP1MΦi assigned to HLA I alleles using the canonical binding motifs according to netMHCpan in the Immune Epitope Database (IEDB) and SYFPEITHI. C) Cumulative percentage of THP1MΦ and THP1MΦi HLA self-peptides within an IC_50_ threshold of 500nM.

The HLA I-bound peptides were nonapeptides (55%, 59%), decapeptides (12%, 13%), octapeptides (7%, 8%), undecapetides (7%, 3%) and duodecapeptides (3%, 5%) in THP1MФ and THP1MФi, respectively **(Fig 3A)**. Thus, in both THP1MФ and THP1MФi the HLA I-bound peptides were dominated by nonapeptides, though the percentage in THP1MФi was slightly higher by 4%. Nonapeptides are the optimum lengths of HLA I-bound peptides [25, 26, 35–37]. The slight increase of nonapeptides in THP1MФi compared to THP1MФ could suggest a shift in antigen processing towards the more optimum peptide lengths for MHC I binding. With 82%, nonapeptides were also the most dominant among the peptides shared between THP1MФ and THP1MФi.

### HLA assignment and binding affinities

The HLA restriction of the peptides was assigned using the netMHCpan in the immune epitope database and the canonical peptide-binding motifs in the SYFPEITHI database [32]. In THP1MΦ the percentage of the peptides identified were in the order HLA-A*02:01 > HLA-B*15:11 > HLA-C*03:03 > unassigned with 35%, 33%, 19% and 13% while in THP1MФi were in the order HLA-B*15:11 > HLA-C*03:03 > HLA-A*02:01 > unassigned with 52%, 20%, 17% and 10%, respectively **(Fig 3B)**. Thus, post infection the percentages of HLA-A*02:01-bound peptides decreased by 18% while those of HLA-B*15:11 increased by 19%; those of HLA-C*03:03 were unaffected. For the HLA-I peptides shared between THP1MΦ and THP1MФi the percentages were in the order HLA-B*15:11 > HLA-A*02:01 > HLA-C*03:03 with 71%, 18% and 12%, respectively. Binding affinity IC_50_ threshold of 500nM has been correlated to immunogenicity [38]. We applied this threshold using the netMHCpan to determine the percentage HLA I peptides (8-14mers) that could stimulate CD8 T cells. The percentages of peptides within this threshold were HLA-A*02:01 (52%, 53%), HLA-B*15:11 (26%, 27%) and HLA-C*03:03 (29%, 47%) in THP1MΦ and THP1MΦi, respectively **(Fig 3C)**. The percentage of HLA-B*15:11 and HLA-A*02:01 peptides that had immune relevance in THP1MΦ and THP1MΦi was approximately the same, despite 19% increase in HLA-B*15:11- and 18% decrease in HLA-A*02:01-bound peptides identified in the infected cells. For HLA-C*03:03, the percentage of peptides that had immune relevance was higher in THP1MΦi (47%) compared to THP1MΦ (29%), though the total percentage of HLA-C*03:03 peptides identified in THP1MΦi and THP1MΦ was about the same.

### The binding motifs for HLA I in THP1MΦ and THP1MΦi

**Fig 4:**
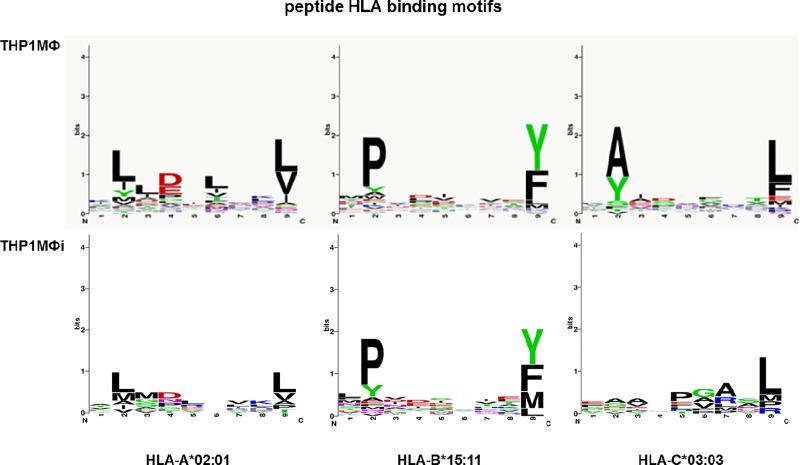
Binding motifs for HLA I-bound self-peptides in THP1MΦ and THP1MΦi. Sequence logos displaying the amino acid preferences for HLA-A*02:01-, HLA-B*15:11- and HLA-C*03:03-bound nonapeptides from THP1MΦ and THP1MΦi.

To determine whether there was a difference in binding motifs of the nonapeptides in THP1MΦ and THP1MΦi, we used sequence logos [33, 34]. In these sequence logos, the height of each column of amino acids is equal to the number of peptide sequences (in bits), and the relative height of each amino acid within each column is proportional to the frequency of the amino acid at that position. The most frequent primary anchor amino acids at position 2 of infected and non-infected THP1MФ HLA-A*02:01-bound peptides were L, and with about equal but lower representation I, Y and M; for the C-terminus these were L and V. For HLA-B*15:11-bound peptides from infected and non-infected cells, P was most prominent at position 2, and Y and F at the C-terminus followed by M in case of the infected cells. For HLA-C*03:03, A and Y were dominant at position 2 of peptides derived from non-infected cells whereas no prominence was found at this position for peptides from infected cells. At the C-terminus of HLA-C*03:03-bound peptides, L was most frequent followed by F in non-infected and M in infected THP1MФ **(Fig 4)**.

### Subcellular locations and biological functions of source proteins

**Fig 5:**
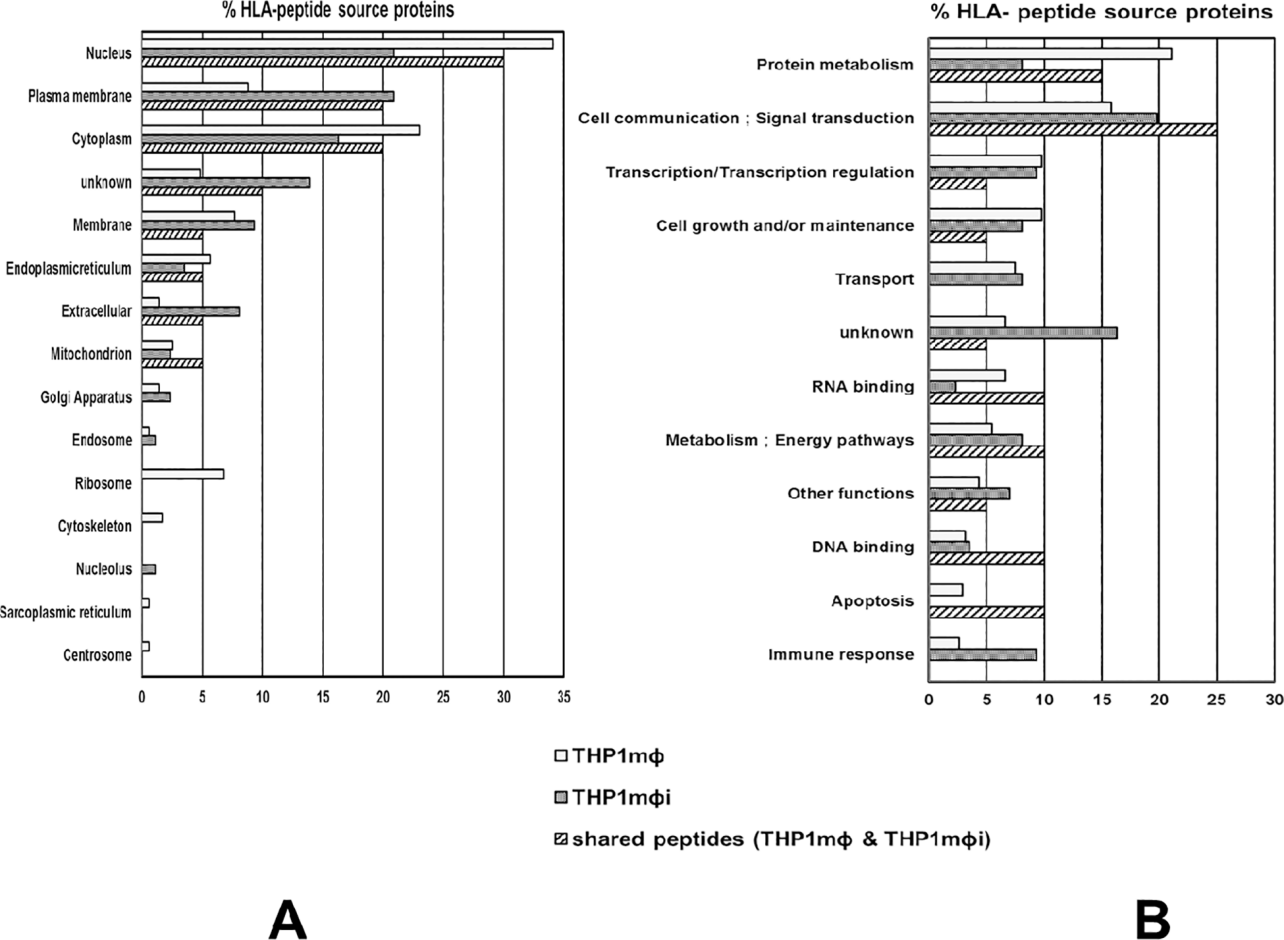
Subcellular locations, biological and molecular functions of the source proteins of the HLA ligands. The subcellular locations, biological and molecular functions of the source proteins of the HLA class I-bound peptides identified by mass spectrometry from THP1MΦ and THP1MΦi were assigned using the Human Protein Reference Database. A) The subcellular locations. B) The biological and molecular functions.

The source proteins of the HLA I-bound peptides from THP1MΦ and THP1MΦi were assigned to the respective subcellular locations and biological functions using the human protein reference database [29]. The subcellular location of the source proteins from THP1MΦ and THP1MΦi were nucleus (34%, 21%), cytoplasm (23%, 16%), plasma membrane (9%, 21%), membrane (8%, 9%), endoplasmic reticulum (6%, 3%), mitochondrion (3%, 2%), extracellular (1%, 8%), endosomes (1%, 1%), and Golgi apparatus (1%, 2%), respectively. For 5% and 14% the subcellular locations were unknown **(Fig 5A)**. In the infected cells, there was thus an increase of source proteins from plasma membrane and extracellular proteins by 12% and 7%, and a decrease in source proteins from nucleus and cytoplasm by 13% and 7%, respectively. In addition, no peptides were identified from source proteins from ribosomes, cytoskeleton and centrosome. The source proteins shared between THP1MФ and THP1MФi were 23% of the total source proteins in THP1MФi and 6% of the total source in THP1MФ and were from almost all subcellular locations in the cell.

The biological functions of the source proteins in THP1MΦ and THP1MΦi were cell communication/signal transduction (16%, 20%), protein metabolism (21%, 8%), transcription/transcription regulation (10%, 9%), transport (7%, 8%), metabolism/energy pathways (5%, 8%), cell growth and/or maintenance (10%, 8%), RNA binding (7%, 2%), immune response (3%, 9%), and DNA binding (3%, 3%), respectively. 7% and 16% of the source proteins had no known biological functions **(Fig 5B).** Post infection the percentage of source proteins involved in immune response, cell communication/signal transduction and metabolism/energy pathways increased by 6%, 4% and 3%, while those involved in protein metabolism, RNA binding, cell growth and/or maintenance decreased by 13%, 5% and 2%, respectively. The source proteins shared between THP1MФ and THP1MФi were mostly involved in cell communications/signal transduction (25%) and protein metabolism (15%).

## Discussion

The total number of HLA class I-restricted self-peptides and source proteins identified from THP1MΦi was four-fold lower compared to those identified in THP1MΦ, and were heterogeneous and individualized. Only a few peptides were found to be shared between the two despite expressing the same HLA alleles.

The strong decrease in the number of HLA class I-restricted peptides from LD-infected THP1MФ has been reproduced in independent experiments and thus not a technical issue, but may rather be due to the following. Firstly, the overall MHC-I expression at the cell surface of THP1MФi was lower compared to THP1MФ including, though not significantly, that of HLA-A*02:01 **(Fig 1B and 1C)**. Reduction of MHC I-restricted antigen presentation upon infection with LD parasites through reduction of MHC I present at the cell surface has also been observed in murine studies albeit no comparative peptidome studies had been done [14, 39]. Though our focus was on HLA I-restricted self-peptides, we also observed lower expression of HLA II by THP1MФi compared to THP1MФ. This observation concurred with murine studies on MHC II, and showed that *Leishmania* inhibits antigen presentation by repressing MHC II expression [14, 39, 40]. Secondly, the infection of THP1MФ by LD resulted in a slight decrease in host cell viability **(Fig 1A**), which might be due to the fact that naturally *Leishmania* promastigotes, upon uptake by macrophages, transform to amastigotes and multiply to eventually rupture the macrophages [2]. Thirdly, although CD83 expression, a marker of macrophage activation, was unchanged in THP1MФi compared to THP1MФ indicating a lack of activation, LD infection resulted in decreased expression of β1 and β2 constitutive proteasome subunits, which could translate into decreased antigen processing efficiency. The impact of proteasome on the quality and quantity of MHC class I ligands had been studied using wild type and proteasome subunits deficient murine dendritic cells [41, 42]: expression of proteasome subunits correlated with increased generation of peptides that are suitable for binding to MHC I molecules.

The heterogeneity and individuality in the HLA I self-peptides and source proteins identified in THP1MФ and THP1MФi depicts differences in protein expression, processing and presentation, as was in other cells and tumor samples [25, 26, 35, 36]. The nonapeptides are the optimum lengths for MHC class I binding [26] and though the infection of THP1MФ by LD did not change the nonapeptides dominance in the identified HLA I-bound peptides, profound differences in antigen processing and presentation were evident, firstly, in the HLA restriction of identified peptides. For THP1MΦ the percentage of the peptides identified for the different HLA-restrictions ranked in the order HLA-A*02:01 > HLA-B*15:11 > HLA-C*03:03 while in THP1MФi they were in the order HLA-B*15:11 > HLA-C*03:03 > HLA-A*02:01. Though HLA-B*15:11 peptides were dominant after infection with LD, only 26% of them were within the IC50 threshold of 500nM. In general, after infection there was a shift of peptides towards lower affinity binders. A previous systematic mapping and characterizing of peptide ligands derived from B*1508, B*1501, B*1503, and B*1510 showed endogenous peptide loaded into B15 to be flexible both in the location of and amino acids at the N-proximal anchors [43]. In addition to this, additional preference of aliphatic amino acids was observed at the C-Terminus after infection, which would, though unconfirmed in Prilliman et al. [43], result in lower binding affinity of the peptides. The differences in antigen processing and presentation were evident in the peptide anchor motifs. For the HLA-A*02:01-bound peptides there were no dominant accessory anchor amino acids at position 6 in THP1MФi compared to the dominant hydrophobic anchor in THP1MФ. For HLA-C*03:03 there was no anchor motifs at position 2 in THP1MФi but a strong preference for A and Y in THP1MФ but a higher percentage of peptides within the IC50 threshold of 500nM in THP1MФi compared to THP1MФ.

In both THP1MΦ and THP1MΦi the peptide source proteins were derived from almost all subcellular locations and were involved in almost all molecular functions of the cells. But differences were observed. Firstly, in THP1MΦi compared to THP1MΦ, there was an increase of source proteins from plasma membrane and extracellular proteins and a decrease in source proteins from nucleus and cytoplasm **(Fig 5A),** and no peptides were identified from ribosomes, cytoskeleton and centrosomes unlike in THP1MΦ. Secondly, in THP1MΦi compared to THP1MΦ, there was an increase of source proteins involved in immune responses, cell communication/signal transduction and metabolism/energy pathways and a decrease in those involved in protein metabolism, RNA binding, cell growth and/or maintenance (**Fig 5B**). The differences in source protein peptide sampling in THP1MΦ and THP1MΦi, would imply differences in protein turnover, as protein turnover correlates with source protein presentation [44, 45]. LD has been shown previously in proteomic studies to globally alter protein expression in THP1 cells [46].

In summary, the infection of macrophages with LD has profound effects on the self-peptide repertoire presented by MHC I molecules, which in parts can be explained with changes in antigen processing including the composition of the proteasomes, and altered protein expression and turn-over in different cellular compartments. In conclusion, the self-displayed by infected macrophages is very different from the self of uninfected cells. This difference may relate to T cell-mediated autoimmune reactions which may explain some of the immune pathology observed in LD patients, as changes in self-peptidome have been shown previously to impact T cell-mediated immune responses [47, 48]. Furthermore, though our focus was on the self-antigens, also the processing of LD antigens may be affected but likely not in the same manner as the self-antigens. LD antigens as exogenous antigensare processed via the MHC class I cross-presentation pathway whereas the self-antigens as endogenous antigens are processed via the classical MHC class I antigen processing and presentation pathway [49, 50]. To check for alterations, a comparison of LD MHC I peptidomes from THP1 derived macrophages incubated with dead LD versus infected with live LD would be required. The results of such studies would however be difficult to compare because dead parasite are expected to be processed through the MHC class II antigen-processing pathway in endolysosomes and the epitopes primarily be presented by MHC class II molecules. Lastly, LD and HIV infect the same host cells, macrophages, but differently, and persist in different subcellular compartments and use different survival mechanisms that affect different key players in the MHC class I antigen processing and presentation pathways [8, 14, 39, 51–54]. LD/HIV co-infection is expected to result in mutual impact on processing and presentation of antigens of both agents. Given the increased fatality of HIV/VL co-infection cases, determination of the effect of LD/HIV co-infection on the self-peptidomes as well as HLA peptidomes of the pathogens, which are yet to be determined, would be vital.

## Supporting information

S1 Table HLA I ligands and source proteins identified from THP1MФi. (PDF)
