# Supplementary material for: Impact of Leishmania donovani infection on the HLA I self peptide repertoire of human macrophages
Source: PLoS One. 2018 Jul 12;13(7):e0200297. doi: 10.1371/journal.pone.0200297 (PMC6042751; doi:10.1371/journal.pone.0200297)
Supplement: S1 Table — (PDF) [file pone.0200297.s001.pdf]

Impact of *Leishmania donovani* infection on the HLA I self-peptide repertoire of antigen-presenting cell line THP1-derived macrophages

Lydon W. Nyambura, Saulius Jarmalavicius and Peter Walden

S1 Table: HLA I ligands and source proteins identified from THP1MΦi.

THP1MΦi

No.	Peptide sequence	Protein name	Measured Mass[M+H]+	Mascot Score	HLA	Subcellular localisation	Biological function
1	SLIMQDDP	Parathyroid hormone 2 receptor OS=Homo sapiens GN=PTH2R PE=1 SV=1 PTH2R_HUMAN	917,4	30	A*02:01	Plasma Membrane	Cell communication ; Signal transduction
2	TLSVPRLP	Plexin-B3 OS=Homo sapiens GN=PLXNB3 PE=1 SV=2 PLXB3_HUMAN	881,58	33	A*02:01	Plasma Membrane	Cell communication ; Signal transduction
3	AVVLGTLHP	EXOSOME COMPLEX COMPONENT RRP46 OS=HOMO SAPIENS GN=EXOSC5 PE=1 SV=1 EXOS5_HUMAN	905,45	22	A*02:01	Nucleolus	Ribonuclease activity
4	KLGNLAKPT	Myelin P2 protein OS=Homo sapiens GN=PMP2 PE=1 SV=3 MYP2_HUMAN	940,55	26	A*02:01	Cytoplasm	Transport
5	TAMDVVYAL	Histone H4 OS=Homo sapiens GN=HIST1H4A PE=1 SV=2 H4_HUMAN	981,49	21	A*02:01	Nucleus	DNA binding
6	SMLDDLRNV*	Integrin beta-2 OS=Homo sapiens GN=ITGB2 PE=1 SV=2 ITB2_HUMAN	1077,47	38	A*02:01	Plasma Membrane	Cell communication ; Signal transduction
7	ALSNLEVKL	Fermitin family homolog 3 OS=Homo sapiens GN=FERMT3 PE=1 SV=1 URP2_HUMAN	985,53	39	A*02:01	Plasma Membrane	unknown
8	LLGPRLVLA	TransMembrane emp24 domain-containing protein 10 OS=Homo sapiens GN=TMED10 PE=1 SV=2 TMEDA_HUMAN	950,59	35	A*02:01	Membrane	Transport
9	TLSDLRVYL	Sulfiredoxin-1 OS=Homo sapiens GN=SRXN1 PE=1 SV=2 SRXN1_HUMAN	1078,57	55	A*02:01	Cytoplasm	Stress response
10	VLMTEDIKL*	Eukaryotic translation initiation factor 4 gamma 1 OS=Homo sapiens GN=EIF4G1 PE=1 SV=4 IF4G1_HUMAN	1076,55	44	A*02:01	Cytoplasm	Protein metabolism
11	YIMGYISKV*	Unconventional myosin-If OS=Homo sapiens GN=MYO1F PE=1 SV=3 MYO1F_HUMAN	1088,51	38	A*02:01	Cytoplasm	Cell growth and/or maintenance

12	VMAPRTLLL*	HLA CLASS I HISTOCOMPATIBILITY ANTIGEN, A-1 ALPHA CHAIN OS=HOMO SAPIENS GN=A PE=1 SV=1 1A01_HUMAN	1028,57	35	A*02:01	Plasma Membrane	Immune response
13	QTMGAPALKI*	Kinesin-like protein KIF13B OS=Homo sapiens GN=KIF13B PE=1 SV=1 KI13B_HUMAN	1044,61	23	A*02:01	Cytoplasm	Cell growth and/or maintenance
14	VYNENLVHMI*	Pre-mRNA-splicing factor SPF27 OS=Homo sapiens GN=BCAS2 PE=1 SV=1 SPF27_HUMAN	1246,55	44	A*02:01	Nucleus	Cell communication ; Signal transduction
15	FPMEIRQY*	Signal transducer and activator of transcription 1-alpha/beta OS=Homo sapiens GN=STAT1 PE=1 SV=2 STAT1_HUMAN	1098,51	41	B*15:11	Cytoplasm	Transcription factor/Transcription factor activity
16	GPQRSVCE	C-TYPE LECTIN DOMAIN FAMILY 4 MEMBER A OS=HOMO SAPIENS GN=CLEC4A PE=1 SV=1 CLC4A_HUMAN	874,29	17	B*15:11	Plasma Membrane	Immune response
17	NAAVVMAV*	AP-3 complex subunit beta-1 OS=Homo sapiens GN=AP3B1 PE=1 SV=3 AP3B1_HUMAN	789,42	25	B*15:11	Plasma Membrane	Protein metabolism
18	DPNLEFVAM*	GTP-binding nuclear protein Ran OS=Homo sapiens GN=RAN PE=1 SV=3 RAN_HUMAN	1050,48	39	B*15:11	Nucleus	Cell communication ; Signal transduction
19	DQMISRIEY*	Casein kinase I isoform alpha OS=Homo sapiens GN=CSNK1A1 PE=1 SV=2 KC1A_HUMAN	1169,54	27	B*15:11	Cytoplasm	Cell cycle
20	FNDPNAKEM*	Zinc finger RNA-binding protein OS=Homo sapiens GN=ZFR PE=1 SV=2 ZFR_HUMAN	1080,46	30	B*15:11	Nucleus	Transcription factor/Transcription factor activity
21	LEGMPQDTY	DNA-binding protein RFX7 OS=Homo sapiens GN=RFX7 PE=1 SV=1 RFX7_HUMAN	1052,6	26	B*15:11	unknown	unknown
22	LPASINTAY	Matrix metalloproteinase-14 OS=Homo sapiens GN=MMP14 PE=1 SV=3 MMP14_HUMAN	948,49	37	B*15:11	Extracellular	Protein metabolism
23	LPREGEQNF	Nck-associated protein 1-like OS=Homo sapiens GN=NCKAP1L PE=1 SV=3 NCKPL_HUMAN	1088,54	17	B*15:11	Plasma Membrane	unknown
24	MPAAGGVLY*	Hepatocyte growth factor-regulated tyrosine kinase substrate OS=Homo sapiens GN=HGS PE=1 SV=1 HGS_HUMAN	893,45	44	B*15:11	Cytoplasm	Cell communication ; Signal transduction
25	MPLEDMNEF*	Replication protein A 32 kDa subunit OS=Homo sapiens GN=RPA2 PE=1 SV=1 RFA2_HUMAN	1156,45	26	B*15:11	Nucleus	DNA binding
26	MPNGTVQRF*	Nucleosome-remodeling factor subunit BPTF OS=Homo sapiens GN=BPTF PE=1 SV=3 BPTF_HUMAN	1064,52	43	B*15:11	Nucleus	Transcription factor/Transcription factor activity
27	NAFKEITTM*	Transcription initiation factor IIB OS=Homo sapiens GN=GTF2B PE=1 SV=1 TF2B_HUMAN	1069,5	34	B*15:11	Nucleus	Transcription factor/Transcription factor activity

28	NPVGGLLEY	Double-stranded RNA-specific adenosine deaminase OS=Homo sapiens GN=ADAR PE=1 SV=4 DSRAD_HUMAN	960,48	37	B*15:11	Nucleus	Deaminase activity
29	SPTDSTPAL	Regulator of cell cycle RGCC OS=Homo sapiens GN=RGCC PE=1 SV=1 RGCC_HUMAN	887,43	29	B*15:11	unknown	unknown
30	SPVDSVLFY	Catenin beta-1 OS=Homo sapiens GN=CTNNB1 PE=1 SV=1 CTNB1_HUMAN	1025,53	37	B*15:11	unknown	unknown
31	TPPTDTILY	Killer cell immunoglobulin-like receptor 3DL1 OS=Homo sapiens GN=KIR3DL1 PE=1 SV=1 KI3L1_HUMAN	1019,54	21	B*15:11	Plasma Membrane	Immune response
32	TPTGIKVVM*	Trafficking protein particle complex subunit 1 OS=Homo sapiens GN=TRAPPC1 PE=1 SV=1 TPPC1_HUMAN	960,52	33	B*15:11	Golgi apparatus	Transport
33	AYVHMVTHF*	Bax inhibitor 1 OS=Homo sapiens GN=TMBIM6 PE=1 SV=2 BI1_HUMAN	1119,49	38	B*15:11	Membrane	Apoptosis
34	FPDKPITQY	Dolichyl-diphosphooligosaccharide--protein glycosyltransferase 48 kDa subunit OS=Homo sapiens GN=DDOST PE=1 SV=4 OST48_HUMAN	1107,54	41	B*15:11	Endoplasmic reticulum	Metabolism ; Energy pathways
35	FPNIPGKSL	Sphingolipid delta(4)-desaturase DES1 OS=Homo sapiens GN=DEGS1 PE=1 SV=1 DEGS1_HUMAN	971,51	51	B*15:11	Endoplasmic reticulum Membrane	Metabolism ; Energy pathways
36	FYMDTSHLF*	Protein PRRC2C OS=Homo sapiens GN=PRRC2C PE=1 SV=4 PRC2C_HUMAN	1175,45	36	B*15:11	Membrane	unknown
37	IYIINVHSM*	DENN domain-containing protein 3 OS=Homo sapiens GN=DENND3 PE=2 SV=2 DEND3_HUMAN	1104,53	39	B*15:11	unknown	unknown
38	KFIDTTSKF	60S ribosomal protein L3 OS=Homo sapiens GN=RPL3 PE=1 SV=2 RL3_HUMAN	1085,32	38	B*15:11	Cytoplasm	Protein metabolism
39	LPDEIYHVY	N-terminal Xaa-Pro-Lys N-methyltransferase 1 OS=Homo sapiens GN=NTMT1 PE=1 SV=3 NTM1A_HUMAN	1147,51	32	B*15:11	Nucleus	Cell cycle
40	LPDTLKVTY	Integrin beta-2 OS=Homo sapiens GN=ITGB2 PE=1 SV=2 ITB2_HUMAN	1048,56	63	B*15:11	Plasma Membrane	Cell communication ; Signal transduction
41	LPIEAPIRM*	Cytochrome c oxidase subunit 2 OS=Homo sapiens GN=MT-CO2 PE=1 SV=1 COX2_HUMAN	1054,55	42	B*15:11	Mitochondrion	Metabolism ; Energy pathways
42	MMNVSKISF*	Sodium-coupled neutral amino acid transporter 2 OS=Homo sapiens GN=SLC38A2 PE=1 SV=2 S38A2_HUMAN	1087,47	58	B*15:11	Plasma Membrane	Transport
43	NAFEVAEKY	Alpha-actinin-4 OS=Homo sapiens GN=ACTN4 PE=1 SV=2 ACTN4_HUMAN	1069,49	41	B*15:11	Cytoplasm	Cell growth and/or maintenance

44	NVIRDAVTY	Histone H4 OS=Homo sapiens GN=HIST1H4A PE=1 SV=2 H4_HUMAN	1049,3	33	B*15:11	Nucleus	DNA binding
45	SPVDSVLFY	Catenin beta-1 OS=Homo sapiens GN=CTNNB1 PE=1 SV=1 CTNB1_HUMAN	1025,46	35	B*15:11	Plasma Membrane	Cell communication ; Signal transduction
46	TYGEIFEKF	NADH dehydrogenase [ubiquinone] 1 subunit C2 OS=Homo sapiens GN=NDUFC2 PE=1 SV=1 NDUC2_HUMAN	1132,51	44	B*15:11	Mitochondrion	Metabolism ; Energy pathways
47	VPLDERIVF	Niban-like protein 1 OS=Homo sapiens GN=FAM129B PE=1 SV=3 NIBL1_HUMAN	1086,57	36	B*15:11	Nucleus	unknown
48	VYAQVARLF	Paired amphipathic helix protein Sin3a OS=Homo sapiens GN=SIN3A PE=1 SV=2 SIN3A_HUMAN	1065,57	41	B*15:11	Nucleus	Transcription factor/Transcription factor activity
49	YPVIMTNEL*	Olfactory receptor 5H6 OS=Homo sapiens GN=OR5H6 PE=2 SV=2 OR5H6_HUMAN	1094,49	31	B*15:11	Plasma Membrane	Cell communication ; Signal transduction
50	FPMTHGNTGF*	Poly(rC)-binding protein 2 OS=Homo sapiens GN=PCBP2 PE=1 SV=1 PCBP2_HUMAN	1123,47	30	B*15:11	Nucleus	RNA binding
51	GPPGQQGTPG	Collagen alpha-2(XI) chain OS=Homo sapiens GN=COL11A2 PE=1 SV=5 COBA2_HUMAN	894,52	16	B*15:11	Extracellular	Cell growth and/or maintenance
52	HPMNDPTRTF*	Lysosomal protein NCU-G1 OS=Homo sapiens GN=C1orf85 PE=1 SV=1 NCUG1_HUMAN	1230,6	15	B*15:11	Membrane	unknown
53	LPSPVTAQKY	Elongation factor 2 OS=Homo sapiens GN=EEF2 PE=1 SV=4 EF2_HUMAN	1102,58	48	B*15:11	Cytoplasm	Protein metabolism
54	MIMQQGMASS*	Mastermind-like domain-containing protein 1 OS=Homo sapiens GN=MAMLD1 PE=1 SV=2 MAMD1_HUMAN	1098,53	16	B*15:11	unknown	unknown
55	ATPVSPAMSY	Inter-alpha-trypsin inhibitor heavy chain H3 OS=Homo sapiens GN=ITIH3 PE=1 SV=2 ITIH3_HUMAN	1022,57	38	B*15:11	Extracellular	Protein metabolism
56	FYMDTSHLF*	Protein PRRC2C OS=Homo sapiens GN=PRRC2C PE=1 SV=4 PRC2C_HUMAN	1176,5	36	B*15:11	RNA binding	Membrane
57	FPASFPNRQY	Minor histocompatibility antigen H13 OS=Homo sapiens GN=HM13 PE=1 SV=1 HM13_HUMAN	1225,55	41	B*15:11	Membrane	Immune response
58	MPVGPDAILRY*	Large proline-rich protein BAG6 OS=Homo sapiens GN=BAG6 PE=1 SV=2 BAG6_HUMAN	1246,59	77	B*15:11	Cytoplasm	Apoptosis
59	NPILSMLTNQTG*	UDP-GlcNAc:betaGal beta-1,3-N-acetylglucosaminyltransferase 2 OS=Homo sapiens GN=B3GNT2 PE=1 SV=2 B3GN2_HUMAN	1303,75	14	B*15:11	Membrane	Metabolism ; Energy pathways

60	YPQQSPYPATGG	Vacuolar protein sorting-associated protein 37C OS=Homo sapiens GN=VPS37C PE=1 SV=2 VP37C_HUMAN	1264,79	19	B*15:11	Endosome	Transport
61	SNEDAADD	Sodium-dependent neutral amino acid transporter B(0)AT2 OS=Homo sapiens GN=SLC6A15 PE=1 SV=1 S6A15_HUMAN	835,36	19	C*03:03	Plasma Membrane	Transport
62	EPPPPPAYR	Thyroid receptor-interacting protein 6 OS=Homo sapiens GN=TRIP6 PE=1 SV=3 TRIP6_HUMAN	1022,51	27	B*15:11	Nucleus	Transcription factor/Transcription factor activity
63	LGVAEALHP	Serine/threonine-protein kinase STK11 OS=Homo sapiens GN=STK11 PE=1 SV=1 STK11_HUMAN	905,45	44	C*03:03	Cytoplasm	Cell communication ; Signal transduction
64	SAAMPGASL*	Beta-1,4-galactosyltransferase 1 OS=Homo sapiens GN=B4GALT1 PE=1 SV=5 B4GT1_HUMAN	819,38	32	C*03:03	Golgi apparatus	Metabolism ; Energy pathways
65	VTVPPGPSL	Putative PIP5K1A and PSMD4-like protein OS=Homo sapiens GN=PIPSL PE=5 SV=1 PIPSL_HUMAN	865,49	27	C*03:03	unknown	unknown
66	YGYTRVAEM*	Matrix metalloproteinase-9 OS=Homo sapiens GN=MMP9 PE=1 SV=3 MMP9_HUMAN	1104,49	46	C*03:03	Extracellular	Protein metabolism
67	EDAELFMAL	Dedicator of cytokinesis protein 5 OS=Homo sapiens GN=DOCK5 PE=1 SV=3 DOCK5_HUMAN	1053,49	40	C*03:03	unknown	Cell communication ; Signal transduction
68	FAEGFVRAL	Transcription factor AP-1 OS=Homo sapiens GN=JUN PE=1 SV=2 JUN_HUMAN	1008,51	55	C*03:03	Nucleus	Transcription factor/Transcription factor activity
69	FVIETARQL	Interferon regulatory factor 2-binding protein-like OS=Homo sapiens GN=IRF2BPL PE=1 SV=1 I2BPL_HUMAN	1075,57	39	C*03:03	Nucleus	Transcription factor/Transcription factor activity
70	SAAFPGASL	DAZ-associated protein 2 OS=Homo sapiens GN=DAZAP2 PE=1 SV=1 DAZP2_HUMAN	819,4	39	C*03:03	unknown	RNA binding
71	MRHTNYSFSP*	Macrophage colony-stimulating factor 1 receptor OS=Homo sapiens GN=CSF1R PE=1 SV=2 CSF1R_HUMAN	1254,5	27	C*03:03	Plasma Membrane	Immune response
72	YVHDAPVRSL	Interleukin-1 beta OS=Homo sapiens GN=IL1B PE=1 SV=2 IL1B_HUMAN	1155,56	42	C*03:03	Extracellular	Immune response
73	TALQQARELQA	TETRATRICOPEPTIDE REPEAT PROTEIN 21B OS=HOMO SAPIENS GN=TTC21B PE=1 SV=2 TT21B_HUMAN	1227,67	11	C*03:03	unknown	unknown
74	YASGRTTGIVM*	Beta-actin-like protein 2 OS=Homo sapiens GN=ACTBL2 PE=1 SV=2 ACTBL_HUMAN	1170,58	32	C*03:03	unknown	unknown

75	GAVLSPSEKSYQ	Interleukin-1 receptor-associated kinase 3 OS=Homo sapiens GN=IRAK3 PE=1 SV=2 IRAK3_HUMAN	1264,78	19	C*03:03	unknown	Cell communication ; Signal transduction
76	NGADGPQGPPGG	Collagen alpha-2(XI) chain OS=Homo sapiens GN=COL11A2 PE=1 SV=5 COBA2_HUMAN	1022,57	17	C*03:03	Extracellular	Cell growth and/or maintenance
77	PGAAKPALAPSFG	Nuclear envelope pore Membrane protein POM 121 OS=Homo sapiens GN=POM121 PE=1 SV=2 P121A_HUMAN	1182,45	30	C*03:03	Nucleus	Transport
78	IILIL	TETRASPANIN-15 OS=HOMO SAPIENS GN=TSPAN15 PE=2 SV=1 TSN15_HUMAN	583,44	23	#	Membrane	Cell communication ; Signal transduction
79	AHLLLL	PROTOCADHERIN GAMMA-A11 OS=HOMO SAPIENS GN=PCDHGA11 PE=1 SV=1 PCDGB_HUMAN	678,5	42	#	Plasma Membrane	Cell growth and/or maintenance
80	TPRAGE	CTD SMALL PHOSPHATASE-LIKE PROTEIN 2 OS=HOMO SAPIENS GN=CTDSPL2 PE=1 SV=2 CTSL2_HUMAN	629,41	24	#	Nucleus	unknown
81	VPKAVM*	Dynamin-1-like protein OS=Homo sapiens GN=DNM1L PE=1 SV=2 DNM1L_HUMAN	659,35	20	#	Cytoplasm	Mitochondrion organization and biogenesis
82	DAGVPGH	Laminin subunit alpha-2 OS=Homo sapiens GN=LAMA2 PE=1 SV=4 LAMA2_HUMAN	651,39	31	#	Extracellular	Cell growth and/or maintenance
83	GIPMVLI*	Rho-related GTP-binding protein RhoF OS=Homo sapiens GN=RHOF PE=2 SV=1 RHOF_HUMAN	757,4	28	#	Plasma Membrane	Cell communication ; Signal transduction
84	PGGAGAS	Neuropeptide B OS=Homo sapiens GN=NPB PE=1 SV=1 NPB_HUMAN	515,3	10	#	unknown	Cell communication ; Signal transduction
85	QSGPEEI	Adenylate cyclase type 8 OS=Homo sapiens GN=ADCY8 PE=1 SV=1 ADCY8_HUMAN	758,4	30	#	Plasma Membrane	Cell communication ; Signal transduction
86	ARSQVVF	Procollagen-lysine,2-oxoglutarate 5-dioxygenase 1 OS=Homo sapiens GN=PLOD1 PE=1 SV=2 PLOD1_HUMAN	805,4	39	#	Endoplasmic reticulum	Metabolism ; Energy pathways
87	TYMLSITG*	Olfactory receptor 6C74 OS=Homo sapiens GN=OR6C74 PE=2 SV=1 O6C74_HUMAN	900,32	21	#	Membrane	Cell communication ; Signal transduction

*amino acid methionine oxidized

HLA assignment undefined
